# Supplementary material for: OptIC-Notch reveals mechanism that regulates receptor interactions with CSL
Source: Development. 2023 Jun 9;150(11):dev201785. doi: 10.1242/dev.201785 (PMC10309584; doi:10.1242/dev.201785)
Supplement: Supplementary information [file develop-150-201785-s1.pdf]

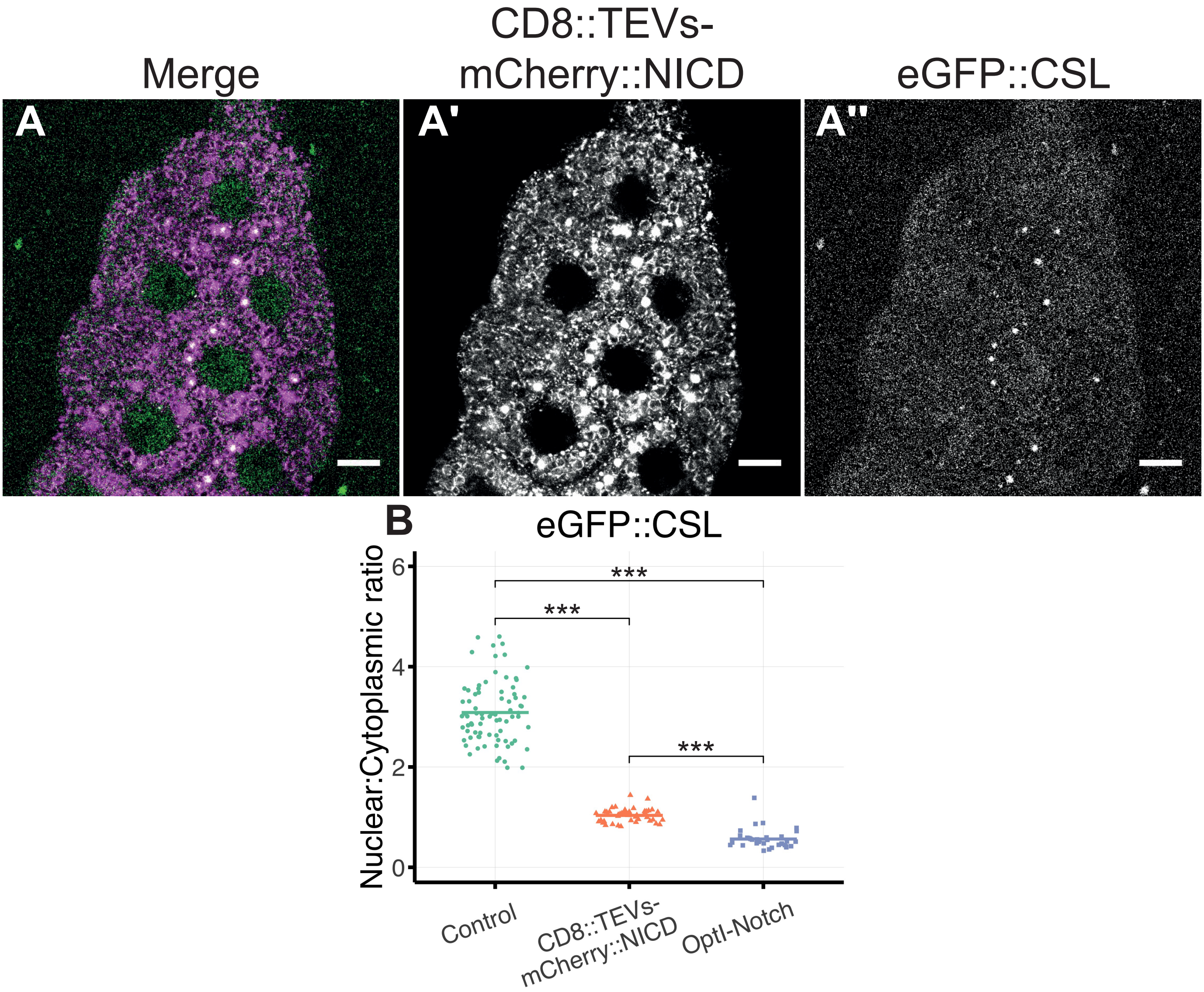

Fig. S1. CSL is sequestered by additional constructs containing NICD.(A) Localisation of eGFP::CSL in salivary glands expressing a construct containing a CD8 transmembrane domain fused to NICD, with a TEV site and mCherry tag in between (magenta and A'). CSL is partially sequestered in the cytoplasm (green and A''). Scale bar = 20 µm.

(B) Nuclear to cytoplasmic ratio of eGFP::CSL in the presence of the CD8 tethered construct and OptI-Notch (without CRY-TEVc), as a control condition, data from flies expressing LacZ and *white* RNAi were used, as shown in Fig S2. Bar represents the mean of ≥7 salivary glands per condition. Welch's two tailed t-test p-value < 0.001.

Development • Supplementary information

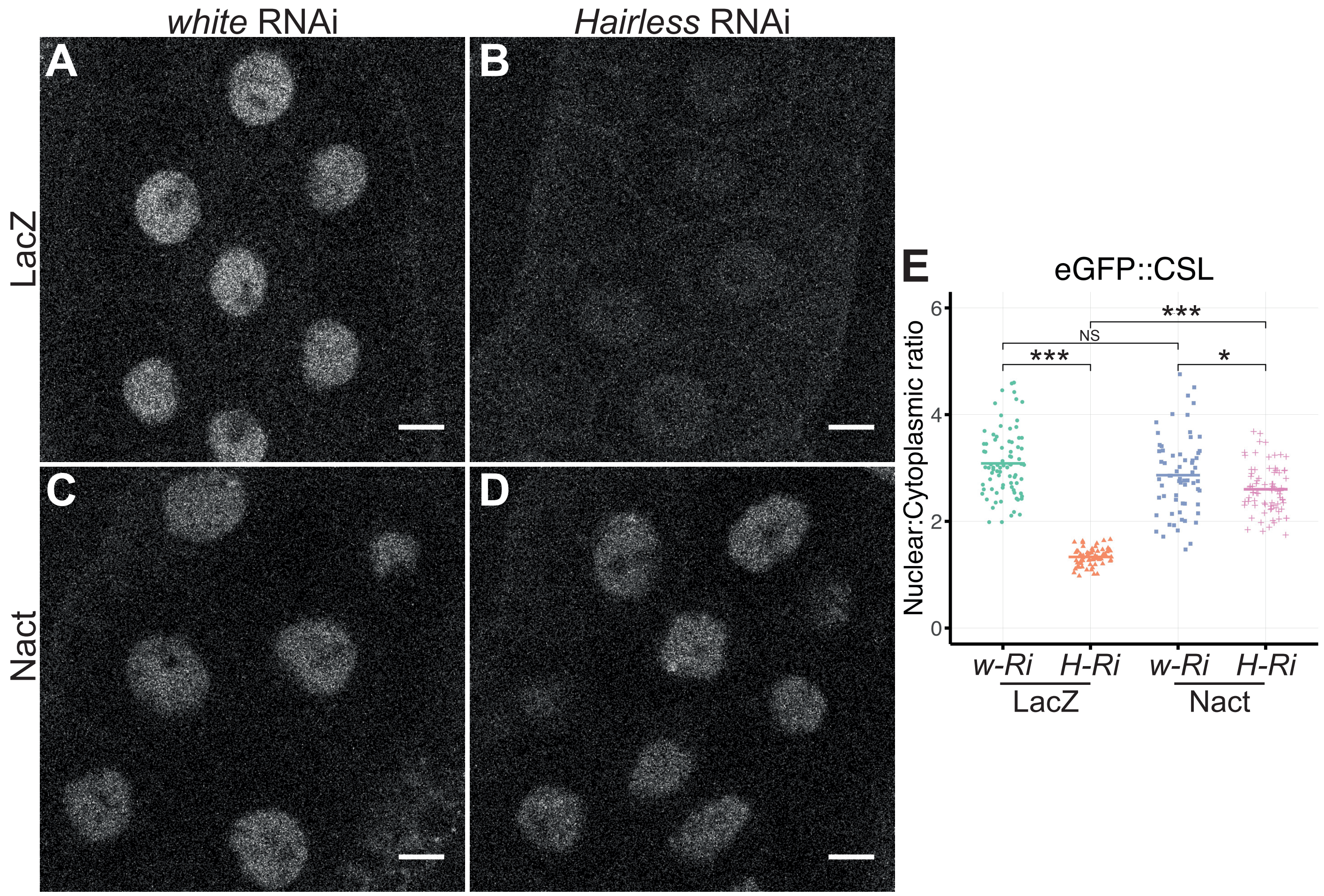

Fig. S2. Effects of Notch on nuclear levels of CSL in the absence of Hairless.(A-D) Localisation of eGFP::CSL in salivary glands expressing LacZ or Nact (the constitutively active Notch, NΔECD) with *white* RNAi (A, C) or *Hairless* RNAi (B, D). A large decrease in CSL levels is seen in the absence of Hairless (B) This decrease in CSL levels is not seen with Nact in the absence of Hairless (D). Scale bar = 20 µm.

(E) Nuclear to cytoplasmic ratio of eGFP::CSL in tissues expressing LacZ or Nact in a *white* or *Hairless* RNAi background. Bars represent the mean of >10 salivary glands per condition. Welch's two tailed t-test p-values for LacZ *w-Ri* vs *H-Ri* < 0.001, Nact *w-Ri* vs *H-Ri* = 0.012, *H-Ri* LacZ vs Nact < 0.001.

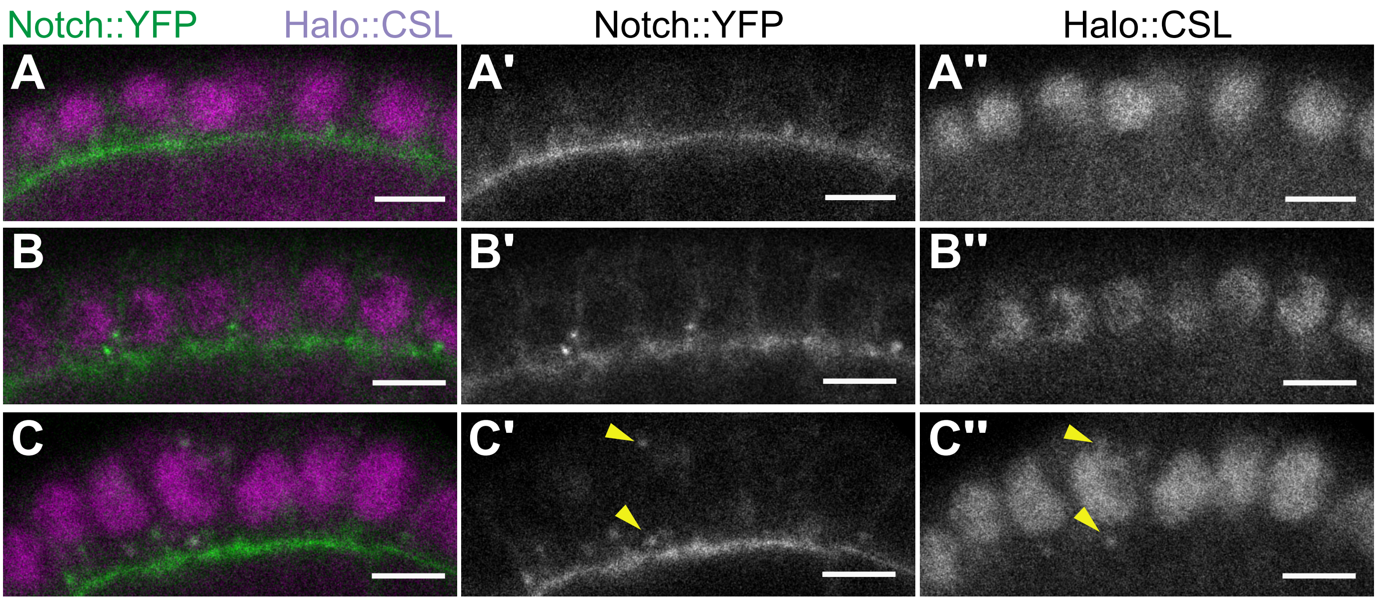

Fig. S3. Notch and CSL do not colocalize at the cell membrane.
(A-C) High-resolution confocal images of stage six egg chambers from *Drosophila* ovaries showing Notch::YFP (A'-C') and CSL::Halo (A''-C'') in the follicle cells. Notch decorates apical and lateral membranes of follicle cells, whereas CSL is restricted to the follicle cell nuclei. In a few cases, co-localization is observed in cytoplasmic puncta (C, yellow arrows in C' and C''). Scale bars = 5 μm.

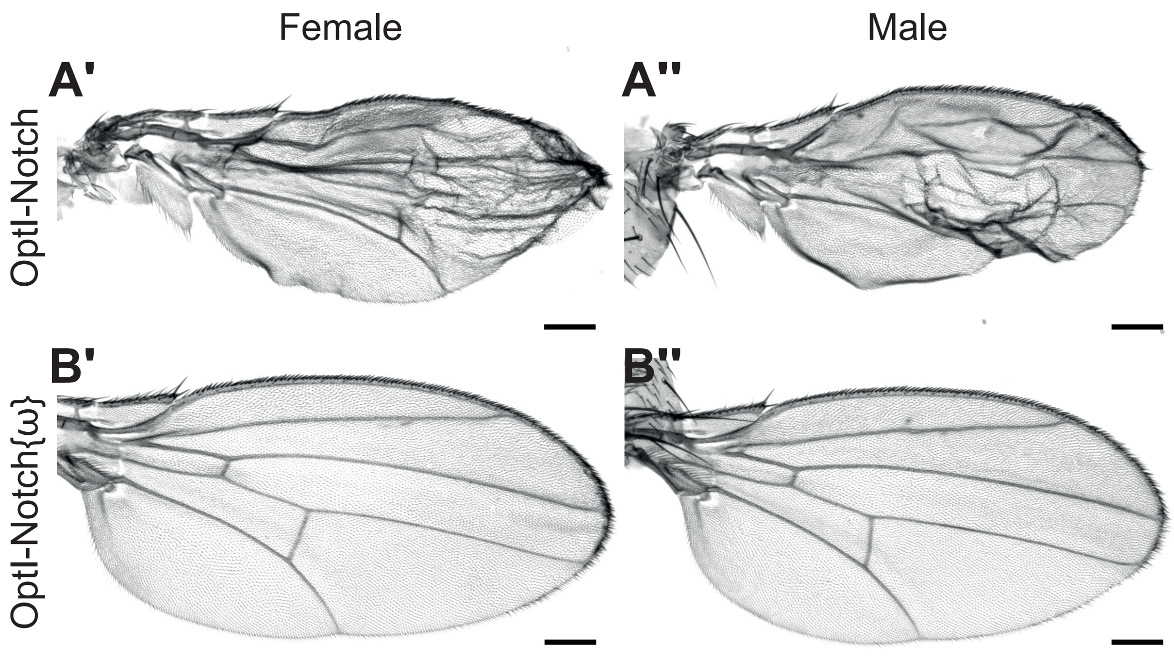

Fig. S4. OptI-Notch{ω} does not generate phenotypes in wings seen with OptI-Notch. OptI-Notch (A) or OptI-Notch{ω} (B) is expressed in the wing in both males and females. Blistering and aberrant vein patterning occurs with OptI-Notch and not OptI-Notch{ω}. Scale bar = 100 μm.

Table S1. Key Resources

REAGENT or RESOURCE	SOURCE	IDENTIFIER
**Chemicals, peptides, and recombinant proteins**		
Shield and Sang M3 insect medium	Sigma Aldrich	Cat #: S3652
Fetal Bovine Serum (Heat inactivated)	Sigma Aldrich	Cat #: F9665
Antibiotic-antimicotic	Gibco	Cat #: 15240062
Poly-L-lysine hydrobromide	Sigma Aldrich	Cat #: P9155
Methyl-cellulose	Sigma Aldrich	Cat #: M0387
Schneider's medium	Biowest	Cat #: L0207-500
Fetal Bovine Serum	Sigma Aldrich	Cat #: F2442
Streptomycin/Penicillin antibiotic mix	Gibco	Cat #: 15140122
Insulin	Sigma Aldrich	Cat #: I5500
Janelia Fluor® 646 HaloTag® Ligand	Promega	Cat #: GA112A
TRI reagent	Invitrogen	Cat #: AM9738
Oligo(dT) primers	Promega	Cat #: C1101
RiboLock RNase inhibitor	Thermo Scientific	Cat #: EO0381
M-MLV reverse transcriptase	Promega	Cat #: M1701
Deoxynucleoside Triphosphate Set	Roche	Cat #: 11969064001
Stellaris RNA FISH Hybridization Buffer	Bioresearch Technologies	Cat #: SMF-HB1-10
Stellaris RNA FISH Wash Buffer A	Bioresearch Technologies	Cat #: SMF-WA1-60
Stellaris RNA FISH Wash Buffer B	Bioresearch Technologies	Cat #: SMF-WB1-20
**Critical commercial assays**		
Ambion's DNA-free DNA removal kit	Invitrogen	Cat #: AM1906
LightCycler 480 SYBR Green I Master PCR kit	Roche	Cat #: 04707516001
**Experimental models: Organisms/strains**		
D. melanogaster 1151-Gal4;;	(Roy and VijayRaghavan, 1997)	Flybase:FBti0007229
D. melanogaster Sal$^{[EPV]}$-Gal4	(Cruz et al., 2009)	Flybase:FBtp0021755. RRID:BDSC_80573
D. melanogaster ;;UAS-*white*-RNAi	Bloomington Drosophila Stock Center	Flybase:FBti0144194. RRID:BDSC_35573
D. melanogaster ;;UAS-*Hairless*-RNAi	Bloomington Drosophila Stock Center	Flybase:FBst0027315. RRID:BDSC_27315
D. melanogaster ;UAS-NΔECD;	(Fortini et al., 1993; Rebay et al., 1993)	N/A
D. melanogaster ;;*E(spl)m8intA*,UAS-ParB1::mCherry	(Gomez-Lamarca et al., 2018)	N/A
D. melanogaster ;;eGFP::CSL	(Gomez-Lamarca et al., 2018)	N/A
D. melanogaster ;;eGFP::CSL[NBM]	(Gomez-Lamarca et al., 2018)	N/A
D. melanogaster ;Notch::YFP;	Courtesy of François Schweisguth (Couturier et al., 2012)	N/A
D. melanogaster ;;Halo::CSL	This paper	N/A
D. melanogaster ;UAS-CRYTEVc (AttP40);	This paper	N/A
D. melanogaster ;UAS-CRYTEVc (AttP51C);	This paper	N/A
D. melanogaster ;;UAS-OptIC-Notch[GFP] (AttP86Fb)	This paper	N/A

D. melanogaster ;;UAS-OptIC-Notch[mCherry] (AttP86Fb)	This paper	N/A
D. melanogaster ;;UAS-OptIC-Notch{ω}[mCherry] (AttP86Fb)	This paper	N/A
D. melanogaster ;UAS-OptIC-Notch{ω}[mCherry] (AttP40);	This paper	N/A
D. melanogaster ;UAS-OptIC-Notch{ω}[mCherry] (AttP40), UAS-CRYTEVc (AttP51C);	This paper	N/A
D. melanogaster ;;UAS-CD8_TEVs_mCherry_NICD (AttP86Fb)	This paper	N/A
**Oligonucleotides**		
Oligonucleotides for mRNA measurements	(Gomez-Lamarca et al., 2018)	Table S1
**Recombinant DNA**		
pCMV-NES-CRY2PHR-TevC	(Lee et al., 2017)	RRID:Addgene_89877
pCMV-TM-CIBN-BLITz1-TetR-VP16	(Lee et al., 2017)	RRID:Addgene_89878
**Software and algorithms**		
ImageJ	(Schneider et al., 2012)	https://imagej.net/ij/index.html
RStudio	(RStudio Team, 2020)	https://posit.co/
**Other**		
R Scripts	This paper	10.5281/zenodo.7607780
HerpNursery II	Lucky Reptile	Model #: CLH501 ASIN: B002NFR0HQ
LED strip lights of 300 5050 RGB LEDs	Nothing Fancy	ASIN: B07Q74XMTQ
Amber book light	LENCENT	ASIN: B082HCTY7W
35 mm Dish, Poly-D-Lysine Coated	MatTek	P35GC-1.5-10-C

Development • Supplementary information

Table S2. Oligonucleotide sequences

**Name**	**Sequence**
E(spl)mβ forward	GCTGGACTTGAAACCGC
E(spl)mβ reverse	AGAAGTGAGCAGCAGCC
RpL32 forward	ATGCTAAGCTGTCGCACAAATG
RpL32 reverse	GTTCGATCCGTAACCGATGT

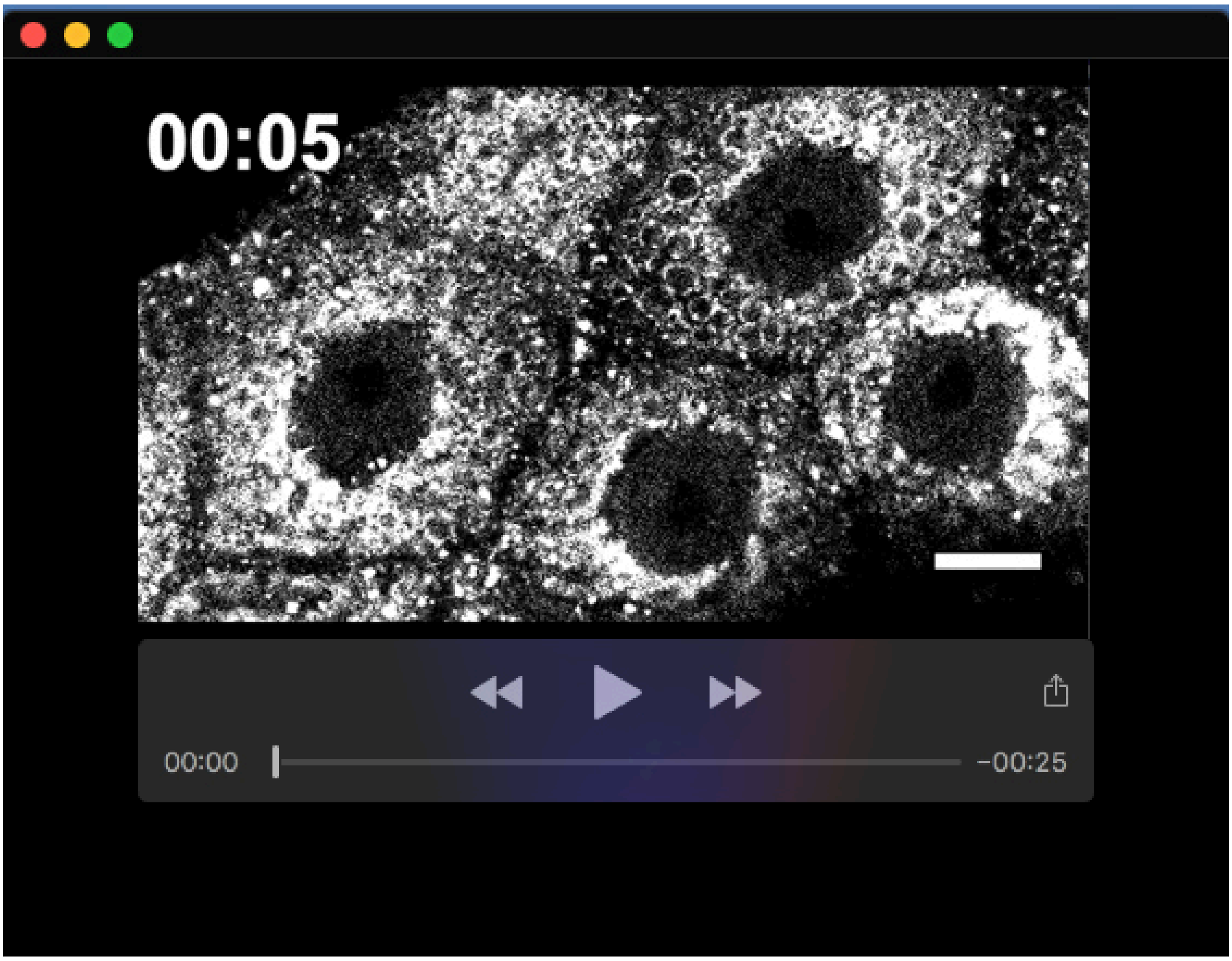

Movie 1. Release of NICD from OptIC-Notch.

OptIC-Notch is activated with 458 nm light for 10 minutes. Release of NICD and its accumulation in the nucleus over time is shown by simultaneous imaging of the mCherry tag. Scale bar = 20 µm.

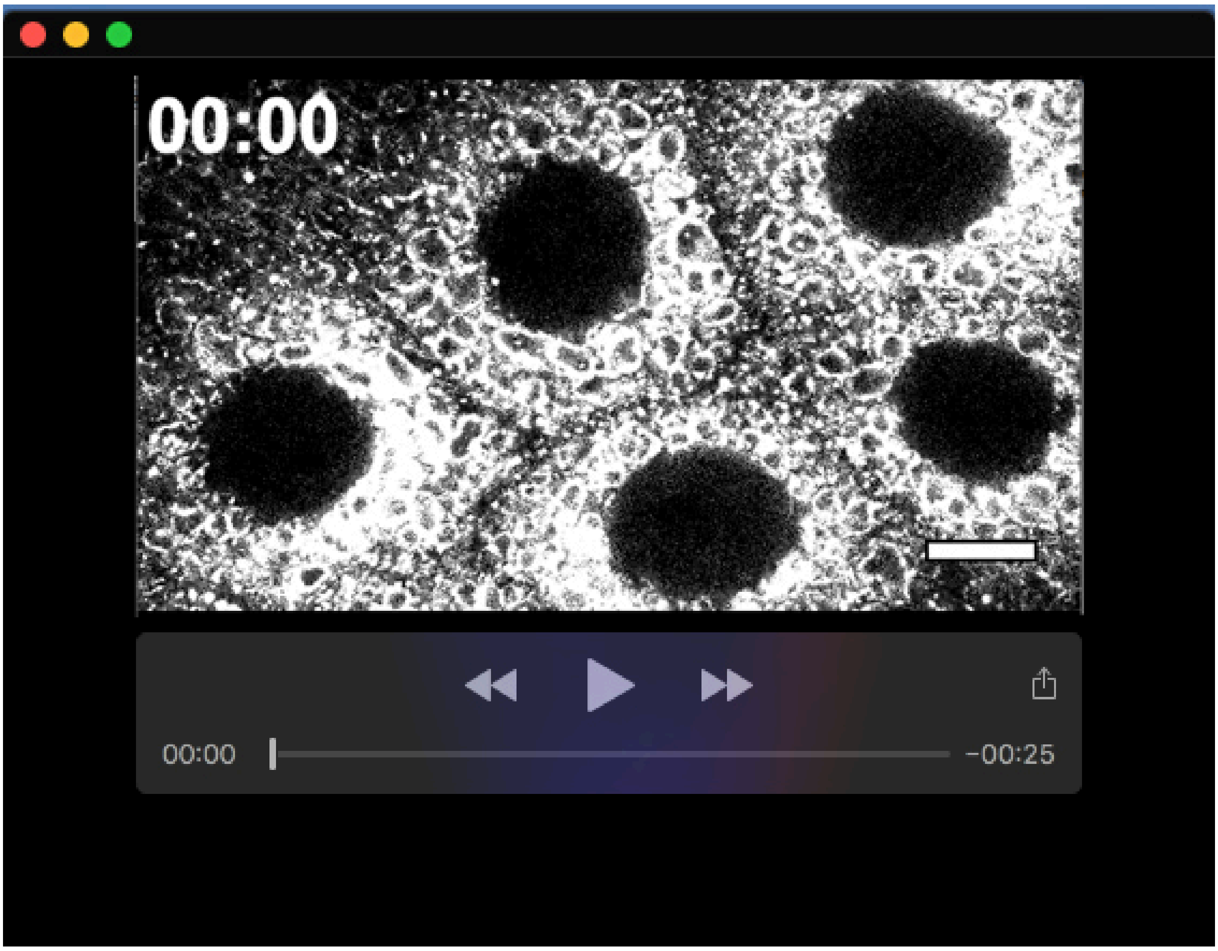

Movie 2. Release of NICD from OptIC-Notch{ω}

OptIC-Notch{ω} is activated with 458 nm light for 20 minutes. Release of NICD and its accumulation in the nucleus over time is shown by simultaneous imaging of the mCherry tag. Scale bar = 20 μm.
