# Supplementary material for: OptIC-Notch reveals mechanism that regulates receptor interactions with CSL
Source: Development. 2023 Jun 9;150(11):dev201785. doi: 10.1242/dev.201785 (PMC10309584; doi:10.1242/dev.201785)
Supplement: Supplementary file 1 [file develop-150-201785_review_history.pdf]

OptIC-Notch reveals mechanism that regulates receptor interactions with CSL

Jonathan M. Townson, Maria J. Gomez-Lamarca, Carmen Santa Cruz Mateos and Sarah J. Bray
DOI: 10.1242/dev.201785

Editor: James Briscoe

Review timeline
Original submission: 21 March 2023
Editorial decision: 29 March 2023
First revision received: 27 April 2023
Accepted: 3 May 2023

Note: This manuscript was transferred to Development after peer review at Current Biology. The author response to the original referee reports from Current Biology was provided to the referees, and used to inform the decision made at Development. The original reports and author response to the original reports has been removed from the Peer Review History.

Original submission

First decision letter

MS ID#: DEVELOP/2023/201785

MS TITLE: OptIC Notch reveals mechanism that regulates receptor interactions with CSL

AUTHORS: Jonathan M. Townson, Maria J. Gomez-Lamarca, and Sarah J. Bray

Thank you for submitting your paper to Development. I have read it along with the previous referees comments that you supplied. I have also asked for input from an expert referee, their comments are appended below, or you can access them online: please go to BenchPress and click on the 'Manuscripts with Decisions' queue in the Author Area.

The overall evaluation is positive and we would like to publish a revised manuscript in Development, provided that the referees' comments can be satisfactorily addressed. Please incorporate the minor edits suggested by the previous Reviewers 1 and 3 and discuss the main caveats raised by Reviewer 2. If you do not agree with any of their criticisms or suggestions explain clearly why this is so. Please send us a point-by-point response indicating your plans for addressing the referee’s comments, and we will look over this and provide further guidance.

Reviewer 1

Advance summary and potential significance to field

This seems like an interesting paper with clear data supporting three main points

1) The authors provide evidence that CSL can bind NIC at the membrane, rather than first encountering NIC in the nucleus, as has been widely assumed. This is interesting and surprising because full length NIC has not previously been found to bind CSL.

© 2023. Published by The Company of Biologists under the terms of the Creative Commons Attribution License (https://creativecommons.org/licenses/by/4.0/).

2) The authors provide evidence that a particular juxtracrine domain is responsible for this binding event. This domain is normally buried in the lipid bilayer of the cell membrane, so this explains why full length Notch has not previously been shown to bind CSL. This therefore resolves apparent contradiction between the authors' findings and previous reports.

3) A technical advance: an efficient optogenetic approach for experimental manipulation of Notch activity.

Comments for the author

This is a nice paper: the data is straightforward, clear, and well quantified. In the interests of a quick decision I've not scrutinised the methods in in great depth but have instead relied quite heavily on the three reviews provided from Curr Biol. Two of the reviewers had no significant concerns, so I paid particular attention to the concerns of reviewer 2. It seems to me that the authors have addressed the concerns of this reviewer satisfactorily in their rebuttal.

The most important concern from reviewer 2, as I see it, is that conclusions are based on overexpressed molecules rather than endogenous molecules. The authors explained clearly why similar manipulations of endogenous molecules would not be feasible (the flies would not survive them) and made a convincing case that the information from the overexpressed constructs was of value.

As I see it, the other concerns are settled to a reasonable degree by clarifications by the authors in their rebuttal.

I note that the Curr Biol editor recommended the minor text edits suggested by reviewers 1 and 3 and include some discussion of the technical caveats outlined by reviewer 2. It seems reasonable to suggest similar conditions for publication in Development. In my view, I think it should be acceptable for the authors to decide which of reviewer 2's 'technical caveats' would be useful to discuss in their revised manuscript, but I'd suggest that it would be useful to include a brief discussion on the rationale for relying on overexpression rather than on modification of endogenous molecules, because this is likely to be a point that arises in many readers minds.

First revision

<u>Author response to reviewers' comments</u>

Reviewer 1 Advance Summary and Potential Significance to Field:
This seems like an interesting paper with clear data supporting three main points
1)The authors provide evidence that CSL can bind NIC at the membrane, rather than first encountering NIC in the nucleus, as has been widely assumed. This is interesting and surprising because full length NIC has not previously been found to bind CSL.
2)The authors provide evidence that a particular juxtracrine domain is responsible for this binding event. This domain is normally buried in the lipid bilayer of the cell membrane, so this explains why full length Notch has not previously been shown to bind CSL. This therefore resolves apparent contradiction between the authors' findings and previous reports.
3)A technical advance: an efficient optogenetic approach for experimental manipulation of Notch activity.

Reviewer 1 Comments for the Author:
This is a nice paper: the data is straightforward, clear, and well quantified. In the interests of a quick decision I've not scrutinised the methods in in great depth but have instead relied quite heavily on the three reviews provided from Curr Biol. Two of the reviewers had no significant concerns, so I paid particular attention to the concerns of reviewer 2. It seems to me that the authors have addressed the concerns of this reviewer satisfactorily in their rebuttal.

© 2023. Published by The Company of Biologists under the terms of the Creative Commons Attribution License (https://creativecommons.org/licenses/by/4.0/).

The most important concern from reviewer 2, as I see it, is that conclusions are based on overexpressed molecules rather than endogenous molecules. The authors explained clearly why similar manipulations of endogenous molecules would not be feasible (the flies would not survive them) and made a convincing case that the information from the overexpressed constructs was of value.

As I see it, the other concerns are settled to a reasonable degree by clarifications by the authors in their rebuttal.

I note that the Curr Biol editor recommended minor text edits suggested by reviewers 1 and 3 and include some discussion of the technical caveats outlined by reviewer 2. It seems reasonable to suggest similar conditions for publication in Development. In my view, I think it should be acceptable for the authors to decide which of reviewer 2's 'technical caveats' would be useful to discuss in their revised manuscript, but I'd suggest that it would be useful to include a brief discussion on the rationale for relying on overexpression rather than on modification of endogenous molecules, because this is likely to be a point that arises in many readers minds.

We thank the reviewer for their positive comments and suggestions. We have made the changes suggested by reviewers 1 and 3 and have significantly revised the text and added new figures to address points made by reviewer 2 as summarized below. In particular, as suggested above, we **have added a caveats section at the end of the conclusion discussing the limitations from the over expression. In addition, we have included a supplementary figure with examples of co-localization under physiological conditions (new Supplementary Figure S3).**

Second decision letter

MS ID#: DEVELOP/2023/201785

MS TITLE: OptIC Notch reveals mechanism that regulates receptor interactions with CSL

AUTHORS: Jonathan Townson, Maria Gomez-Lamarca, Carmen Santa Cruz Mateos, and Sarah Bray
ARTICLE TYPE: Research Report

I am happy to tell you that your manuscript has been accepted for publication in Development, pending our standard ethics checks.

Reviewer 1

Advance summary and potential significance to field

.

Comments for the author

The authors have now addressed concerns of all reviewers to a satisfactory degree. They have included a useful "caveats" section in which they discuss their reliance on overexpressed molecules. I have no further concerns. Congratulations to the authors on this nice study.

© 2023. Published by The Company of Biologists under the terms of the Creative Commons Attribution License (https://creativecommons.org/licenses/by/4.0/).
